# Direct tumour–adipocyte contact / Stroma AReactive Invasion Front Area (TAC/SARIFA) predicts adverse outcome in colorectal cancer: a focused evidence synthesis and meta‐analysis

**DOI:** 10.1111/his.70237

**Published:** 2026-07-16

**Authors:** Nic G Reitsam, Bruno Märkl, Iris D Nagtegaal, Magali Svrcek

**Affiliations:** ^1^ Pathology, Faculty of Medicine University of Augsburg Augsburg Germany; ^2^ Bavarian Cancer Research Center (BZKF) Augsburg Germany; ^3^ Department of Pathology Radboud University Medical Center Nijmegen The Netherlands; ^4^ Department of Pathology Léon Bérard Comprehensive Cancer Center Lyon France; ^5^ Sorbonne Université, LIMICS, INSERM UMRS 1142 Paris France

## Abstract

**Aims:**

Direct tumour‐adipocyte interaction at the invasion front, termed Stroma AReactive Invasion Front Areas (SARIFA), has recently emerged as a novel H&E‐based biomarker in colorectal cancer (CRC). SARIFA positivity is defined by a direct tumour–adipocyte contact (TAC) without intervening stroma or inflammatory infiltrate. We performed a focused evidence synthesis of all published studies and a meta‐analysis to summarize the evidence on the prognostic value of TAC/SARIFA in CRC.

**Methods and Results:**

Eligible studies, identified via PubMed (7 May 2026), were observational cohorts reporting multivariable‐adjusted hazard ratios (HRs) for TAC/SARIFA positivity vs negativity, with cancer‐specific survival (CSS) and overall survival (OS) as endpoints. All included studies were retrospective in design. Study quality was assessed using the Newcastle–Ottawa Scale (NOS). Adjusted HRs were pooled using random‐effects meta‐analysis with restricted maximum‐likelihood estimation and Hartung–Knapp confidence intervals. Of 17 records identified, 12 CRC‐focused original publications were included in the qualitative synthesis. Seven cohorts (5,932 patients) contributed CSS and five cohorts (4,545 patients) OS data, drawn from eight independent cohorts across six studies. All studies were rated good quality (NOS 7–9/9). TAC/SARIFA positivity was associated with inferior CSS (pooled HR 1.77, 95% confidence interval [CI] 1.55–2.01) and OS (pooled HR 1.53, 95% CI 1.39–1.68), with no relevant heterogeneity detected.

**Conclusion:**

TAC/SARIFA positivity is independently associated with inferior CSS and OS in CRC, warranting prospective validation. We propose TAC as a more descriptive terminology.

## Graphical abstract

TAC/SARIFA, defined by direct tumour–adipocyte contact at the invasion front, represents a reproducible, biologically meaningful H&E biomarker with consistent adverse prognostic associations in CRC.

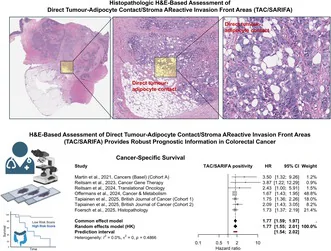

**Abbreviations**
CI: confidence interval
CMS: consensus molecular subtypes
CRC: colorectal cancer
CSS: cancer‐specific survival
DR: desmoplastic reaction
DSS: disease‐specific survival
EMT: epithelial‐mesenchymal transition
GMS: Glasgow Microenvironment Score
HR: hazard ratio
NOS: Newcastle–Ottawa Scale
OS: overall survival
PDS: pathway‐derived subtype
SARIFA: Stroma AReactive Invasion Front Areas
TAC: tumour–adipocyte contact
TAF: tumour‐adipose feature
TME: tumour microenvironment

## Introduction

Tumour–host interactions at the invasive front are increasingly recognized as key determinants of colorectal cancer (CRC) progression and patient outcomes.^1^ Beyond tumour‐intrinsic features, the local tumour microenvironment (TME) critically modulates invasion, immune evasion and therapeutic response.^2^, ^3^ However, few TME and invasion front biomarkers have proven sufficiently robust and practical for routine histopathology.

Among invasion front biomarkers, tumour budding, which captures discohesive clusters of tumour cells and is considered to be the morphological correlate of epithelial–mesenchymal transition (EMT),^4^, ^5^ has already been incorporated into routine pathology reporting,^6^ though substantial interobserver variability limits its clinical impact.^7^, ^8^, ^9^ Other microenvironment‐based markers, such as the Glasgow Microenvironment Score (GMS)^10^, ^11^ and desmoplastic reaction (DR) patterns,^12^, ^13^, ^14^ show robust prognostic value but lack clinical implementation.

Recently, direct tumour–adipocyte contact (TAC) at the invasion front at so‐called Stroma AReactive Invasion Front Areas (SARIFA) has emerged as a novel, histomorphological biomarker assessable on routine H&E sections.^15^ SARIFA captures direct physical interaction between invading tumour cells and adjacent adipocytes without intervening stroma or inflammatory infiltrate, reflecting a distinct tumour–host interaction pattern.^15^, ^16^, ^17^, ^18^, ^19^, ^20^, ^21^, ^22^, ^23^, ^24^, ^25^, ^26^, ^27^, ^28^, ^29^, ^30^, ^31^ Multiple cohort studies have validated SARIFA's independent prognostic value in CRC and investigated its underlying biology,^16^, ^17^, ^18^, ^19^, ^20^, ^21^, ^22^, ^23^, ^24^, ^25^, ^27^, ^32^ although these studies vary in cohort composition, endpoints and statistical approaches. TAC/SARIFA positivity is predominantly observed within subserosal adipose tissue, resulting in very low positivity rates in early‐stage (pT1/2) tumours across all reported cohorts.^15^

Therefore, this study provides a structured synthesis of published SARIFA studies in CRC, emphasizing consistency of prognostic effects across cohorts and endpoints. We propose the term TAC as a more descriptive and clearer designation while maintaining TAC/SARIFA for consistency with existing literature in the current paper.

## Methods

### Histopathological Definition and Reproducibility of TAC/SARIFA

The following section defines TAC/SARIFA as used across the included studies and summarizes the available evidence on assessment reproducibility: TAC/SARIFA positivity is defined as an area at the tumour invasion front, in which at least one tumour gland or a group of at least five (≥5) tumour cells are directly adjacent to adipocytes without interjacent inflammatory infiltrate, or without an intervening desmoplastic stromal reaction. Even if only a single TAC/SARIFA is present, for example, a single tumour gland surrounded directly by adipose tissue, the case is classified as TAC/SARIFA‐positive.

Representative examples of TAC/SARIFA‐positive and TAC/SARIFA‐negative CRCs are displayed in Figure 1.

**Figure 1:**
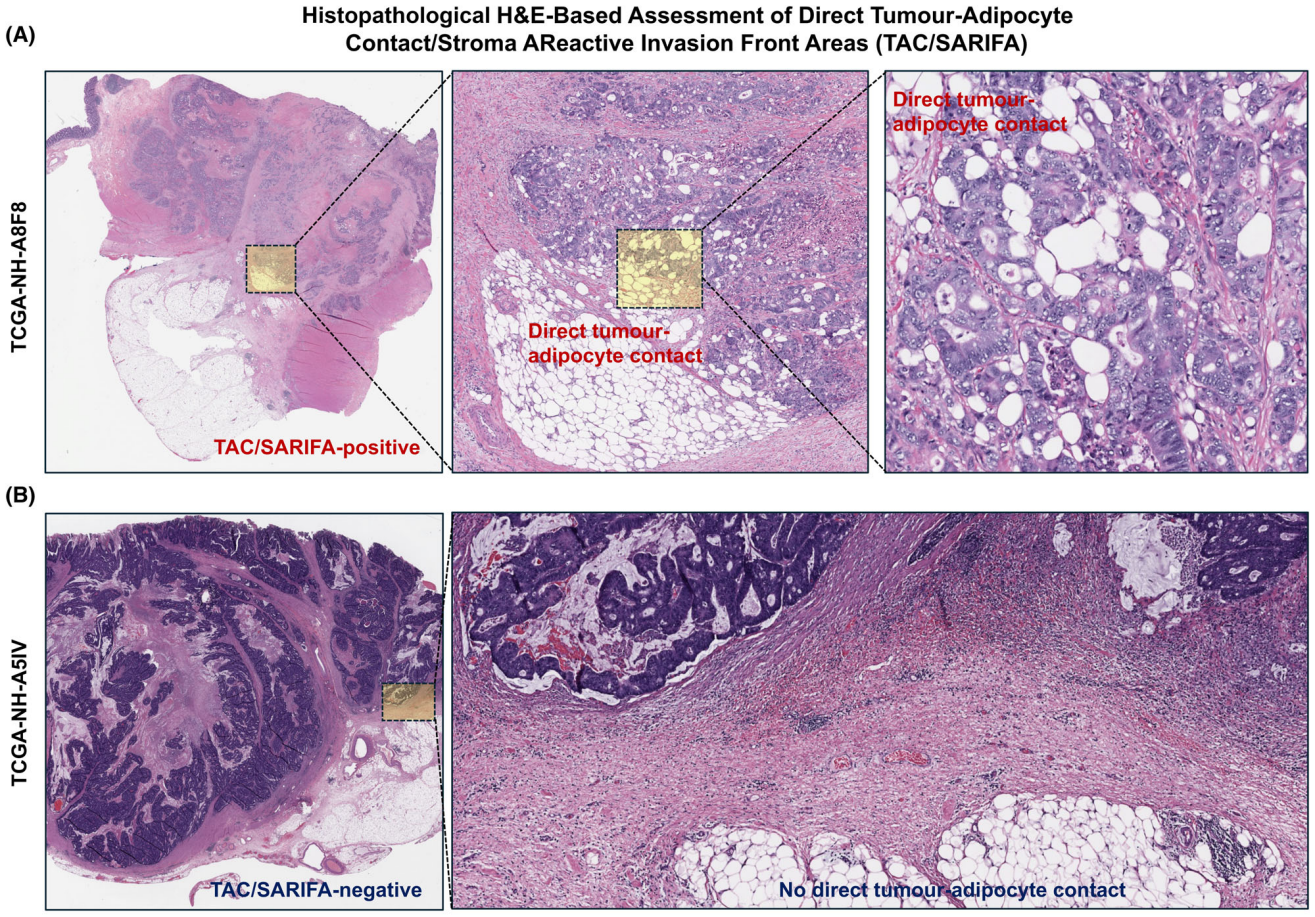
Representative examples of TAC/SARIFA‐positive and TAC/SARIFA‐negative CRCs of the TCGA cohorts. TAC/SARIFA status is solely assessed on H&E slides. **(A)** TAC/SARIFA positivity is defined by direct tumour–adipocyte interaction without intervening stroma or inflammatory infiltrate. **(B)** TAC/SARIFA negativity is defined by the absence of direct tumour–adipocyte interaction. Both whole slide images were accessed via GDC portal (https://portal.gdc.cancer.gov/, 30 December 2025). For additional examples on TAC/SARIFA‐positive and TAC/SARIFA‐negative CRCs, we refer to the initial studies all showing numerous representative examples. The SARIFA assessment of the publicly available TCGA cohorts COAD and READ has also been published alongside our publication on this,^20^ and the digitized slides can serve here as training material for interested pathologists and also enable further downstream analyses. Previous studies used the term SARIFA; we propose the dual terminology tumour–adipocyte contact (TAC)/SARIFA, with TAC being more descriptive of the underlying biological phenomenon.

In routine practice, TAC/SARIFA can, of course, be assessed on all available tumour‐containing slides. In research settings, sometimes only one representative slide may be available.

TAC/SARIFA assessment shows a low interobserver variability with good to very good kappa values across different cohorts and settings. Reported kappa values range from 0.77 to 0.917.^22^, ^23^, ^25^ Kappa values in CRC so far exceed those for TAC/SARIFA assessment in other epithelial carcinomas, like gastric or pancreatic cancer.^28^, ^30^

### Ethical Approval

As this study exclusively analysed previously published data, no ethical approval or informed consent was required.

### Literature Identification, Study Selection & Eligibility Criteria

The review was not prospectively registered. The evidence synthesis was structured around the following framework: Population—patients with CRC (colon and/or rectal cancer, as defined in each original study); Exposure—TAC/SARIFA positivity (as assessed on H&E‐stained sections); Comparator—TAC/SARIFA negativity; Outcomes—cancer‐specific survival (CSS) and overall survival (OS) as primary endpoints; Study type—observational cohort studies (reporting multivariable‐adjusted hazard ratios). Studies including patients with colorectal cancer (colon and/or rectal cancer, as defined in the original studies) were considered eligible.

This study was conducted as a focused evidence synthesis and meta‐analysis rather than a conventional systematic review. We nevertheless aligned our reporting with PRISMA 2020^33^ and MOOSE^34^ recommendations where applicable; a completed PRISMA 2020 checklist is provided as Table S1.

A structured literature search was performed in PubMed on 7 May 2026 using the search terms: (SARIFA OR “Stroma AReactive Invasion Front Areas”) AND (“colorectal cancer” OR “colon cancer” OR “rectal cancer” OR “colorectal carcinoma” OR “colon carcinoma” OR “rectal carcinoma”). No restrictions on publication date were applied. The first author, NGR, performed the literature search.

Given the still‐limited and emerging literature on TAC/SARIFA, all identified publications in this field were already known to the author group and were systematically re‐screened through the formal database search strategy.

Study selection is summarized in a PRISMA‐style 2020 flow diagram (Figure S1). The database search identified 17 records, all of which were screened at the abstract and full‐text level; one was focusing on pancreatic adenocarcinoma^28^ and on oesophagogastric cancer,^29^ and three were reviews.^15^, ^35^, ^36^ At the time of retrieval, PubMed additionally displayed several of the SARIFA‐focused review articles (*n* = 3) through automatic indexing and relevance matching, despite not strictly matching all components of the Boolean query. Of the remaining 12 original articles,^16^, ^17^, ^18^, ^19^, ^20^, ^21^, ^22^, ^23^, ^24^, ^25^, ^27^, ^32^ 6 studies comprising eight independent, non‐overlapping CRC cohorts^18^, ^20^, ^22^, ^23^, ^25^, ^27^ reported adjusted hazard ratios for survival endpoints and were therefore eligible for quantitative synthesis (meta‐analysis, Table 1). The remaining reports (*n* = 6, Table 2)^16^, ^17^, ^19^, ^21^, ^24^, ^32^ were excluded from meta‐analysis because they (i) investigated mixed tumour entities (e.g., gastrointestinal cohorts including CRC and gastric cancer without CRC‐stratified survival estimates^16^), and/or (ii) focused on biological associations of TAC/SARIFA without reporting time‐to‐event outcomes or multivariable HRs^16^, ^17^, ^21^, ^24^, ^32^ and/or (iii) were based on overlapping cohorts (NLCS^19^). These studies were nevertheless included in the qualitative synthesis to provide a comprehensive overview of TAC/SARIFA‐related biology and clinicopathological correlates.

**Table 1:** Overview of studies on TAC/SARIFA in colorectal cancer contributing to meta‐analysis

First author(s)	Year	Journal	Country	Study design	Cohort size Total	CRC stages	Entities	Age in years (median [min‐max])	Sex distribution	Type of outcome data (all)	Median follow‐up in years	Events	Covariates in a multivariable model	TAC/SARIFA positivity rate	Main findings
Martin	2021	Cancers (Basel)	Germany	Retrospective, single centre	449 (Cohort A: 196, Cohort B: 253)	II‐III	Colon	Cohort A: 71 [64–79] Cohort B: 71 [61–77]	Cohort A: female 42%, male 58% Cohort B: female 44%, male 56%	Overall survival, cancer‐specific survival, metastasis‐free survival	Cohort A: 6.4 (CSS) Cohort B: 4.2 (OS)	Cohort A: 23/196 Cohort B: 68/253	Age, pT, pN, grading, vascular invasion, lymphatic vessel invasion, tumour budding, MSS, sidedness, SARIFA	Cohort A: 16% Cohort B: 31%	25% of all tumours are TAC/SARIFA‐positive association with adverse features kappa‐value: 0.77 & 0.87 associated with poor prognosis
Reitsam	2023	Cancer Gene Therapy	USA [TCGA cohorts]	Retrospective, multi‐centre	207	I‐IV	CRC	67 [31–90]	Female 50%, male 50%	Overall survival, disease‐specific survival, progression‐free survival	26.4 months ~2.2 years	Not explicitly reported in the original publication	For OS: metastasis, AJCC, age, SARIFA For CSS: metastasis, AJCC, SARIFA	33.3%	TAC/SARIFA‐positivity associated with poorer overall, disease‐specific and progression‐free survival distinct biology with upregulation of lipid metabolism, immunologic and stromal changes (CMS1 & 4) potential differential drug sensitivity (OncoPredict)
Reitsam	2024	Translational Oncology	Germany	Retrospective, single centre	164	I‐IV	CRC	69 [45–88]	Female 57%, male 43%	Recurrence‐free survival, cancer‐specific survival	6.0 TAC/SARIFA‐positive / 4.8 TAC/SARIFA‐negative (combined not reported)	CSS events: 26	pT, pN, grade, proportion of tumour, SARIFA	22.6%	Association with adverse features and poor survival outcomes TAC/SARIFA‐positivity, measured at the invasive front, was linked to higher stroma content and lower vessel density at the luminal tumour surface
Offermans*, Reitsam*	2024	Cancer & Metabolism	Netherlands	Population‐based series	1727	I‐IV	CRC	74 [55–89]	Female 45%, male 55%	Overall survival, cancer‐specific survival	4.8 (restricted to 10 years for survival analysis)	CSS: 723/1,727 OS: 1,128/1,727	Age, sex, location, pTNM, grade, adjuvant therapy, MMR	22.0%	Association with adverse features TAC/SARIFA‐positivity is more common in the Warburg‐high subtype link to poor survival outcomes
Tapiainen	2025	British Journal of Cancer	Finland	Retrospective, two centres	1876	I‐IV	CRC	Age < 65, 65–75, >75 Cohort 1: 26%, 35%, 39% Cohort: 2: 30%, 37%, 33%	Cohort 1: female 51%, male 49% Cohort 2: female 47%, male 53%	Cancer‐specific survival, overall survival	Cohort 1: 10 Cohort 2: 7.0 (both limited to 10)	CSS: C1: 296/1,063 C2: 155/771 OS: C1: 531/1,063 C2: 284/771	Sex, age, year of operation, tumour location, AJCC stage, grade, lymphovascular invasion, tumour budding, MMR status, BRAF status	Cohort 1: 30% Cohort 2: 31%	Association with poor survival outcomes and adverse features TAC/SARIFA‐positivity linked to immune dysregulation, e.g. lower numbers of CD3+ T cells and higher densities of M2‐like macrophages
Foersch	2025	Histopathology	Germany	Retrospective, three centres	1298	II‐IV	CRC	71 [not reported]	Female 43%, male 57%	Overall survival, disease‐specific survival, disease‐free survival	Not explicitly reported in the original publication, censored at 10	Not explicitly reported in the original publication	pT, pN, pM, resection status, tumour budding, histopathological subtype, WHO grade, gender, age, tumour localisation	41.1%	Association with adverse features linked to poor prognosis additional prognostic value within tumour budding and grade categories

CRC, colorectal cancer; CSS, cancer‐specific survival; MMR, mismatch repair (status); MSS, microsatellite stability (status); OS, overall survival; SARIFA, Stroma AReactive Invasion Front Areas; TAC, tumour–adipocyte contact.

**Table 2:** Overview of studies on TAC/SARIFA (not contributing to meta‐analysis)

First author(s)	Year	Journal	Country	Study design	Cohort size total	CRC stages	Entities	Age (median [min‐max])	Sex distribution	Type of outcome data	TAC/SARIFA positivity rate	Main findings
Reitsam	2023	Cancers (Basel)	Germany	Retrospective, single centre	45	I‐IV	CRC	66 [42–84]	Female 60%, male 40%	/	33.3%	Natural Killer (NK) cells are reduced in the peripheral blood of TAC/SARIFA‐positive cases CD56‐ and CD57‐positive NK/NK‐like cells are decreased at SARIFAs
Hu	2023	Cancer Research Communications	China	Retrospective, multi centre	620	I‐IV	CRC	Not described for the subset with available TAC/SARIFA assessment	Not described for the subset with available TAC/SARIFA assessment	Not described for the subset with available TAC/SARIFA assessment	19%	Immature and middle desmoplastic reactions were associated with TAC/SARIFA‐positivity.
Reitsam	2024	Communications Medicine	Germany/USA	Retrospective, two cohorts	/	I‐IV	CRC	TCGA‐CRC, see Reitsam *et al*., Cancer Gene Therapy	TCGA‐CRC, see Reitsam *et al*., Cancer Gene Therap	Overall survival, disease‐specific survival, progression‐free survival (no multivariable analysis)	/	Overlap between deep learning‐derived tumour adipose feature (TAF) and human‐derived TAC/SARIFA
Reitsam	2025	British Journal of Cancer	Germany/USA	DFCI Orion cohort, retrospective	35	I‐IV	CRC	Not reported (correspondence)	Not reported (correspondence)	Overall survival, progression‐free survival	49%	Association with immunologic changes
Reitsam*, Grosser*	2026	Pathobiology	Germany	Retrospective, single centre	96	I‐IV	CRC	71 [27–88]	Not reported (correspondence)	Overall survival (no multivariable analysis)	36.5%	Association with upregulation of the uPA/uPAR system and matrix metalloproteinases

CRC, colorectal cancer; SARIFA, Stroma AReactive Invasion Front Areas; TAC, tumour–adipocyte contact.

### Systematic Synthesis, Meta‐Analysis and Statistical Testing

For each study/cohort, we extracted the adjusted hazard ratio (HR) with 95% confidence interval (CI) from multivariable Cox proportional hazards models for OS and CSS (including disease‐specific survival [DSS] where reported). Additional endpoints (recurrence‐free survival, progression‐free survival, and metastasis‐free survival) were summarized descriptively but were not pooled due to limited cohort numbers and endpoint heterogeneity. These endpoints were available in single cohorts only (recurrence‐free survival: 1 cohort, *n* = 164^27^; progression‐free survival: 1 cohort, *n* = 207^20^; metastasis‐free survival: 1 cohort, *n* = 253^25^).

Primary outcomes were CSS and OS, for which adjusted hazard ratios from multivariable Cox models were pooled. Secondary outcomes, summarized descriptively, included recurrence‐free survival, progression‐free survival, metastasis‐free survival and clinicopathological associations (including tumour stage, grade, budding and molecular features).

Random‐effects meta‐analyses were conducted separately for OS and CSS/DSS using inverse‐variance weighting. Between‐study heterogeneity was assessed using Cochran's *Q*, *I* ^2^ and *τ* ^2^. Confidence intervals were computed using the Hartung–Knapp method, and prediction intervals were reported to reflect expected effect variation across settings.

Formal assessment of publication bias was not performed because the limited number of eligible studies precludes a reliable evaluation.

Analyses were performed in R using the *meta* package; forest plots display HRs on the original scale (back‐transformed from log scale) with random‐effects summary diamonds and prediction intervals.

Additionally, TAC/SARIFA positivity frequency (%) and the number of scored cases were extracted for each cohort. To account for differences in cohort size, we additionally calculated a case‐weighted mean frequency, weighting each cohort's TAC/SARIFA percentage by its number of scored cases.

Study quality for all studies included in the meta‐analysis was assessed using the Newcastle‐Ottawa Scale (NOS) for cohort studies, covering the domains of selection, comparability and outcome (Table S2). Additionally, a descriptive assessment of study quality across all 12 publications was performed (Table S3).

## Results

We employ the dual terminology TAC/SARIFA throughout this article to bridge the established acronym from prior publications (SARIFA) with the more descriptive designation TAC, which better captures the underlying morphological and biological phenomenon.

### Characteristics of Included Studies

TAC/SARIFA status was established in 12 different retrospective and/or population‐based CRC cohorts (in some studies, more than one cohort).^16^, ^17^, ^18^, ^19^, ^20^, ^21^, ^22^, ^23^, ^24^, ^25^, ^27^, ^32^

These retrospective cohorts ranged from early‐stage to advanced disease and included single‐centre, multicentre or population‐based studies, encompassing patients across Europe and the United States.

Across 12 cohorts comprising 6475 colorectal cancer cases, TAC/SARIFA positivity ranged from 16% to 49% (median 31%; case‐weighted mean 29%, Figure 2).

**Figure 2:**
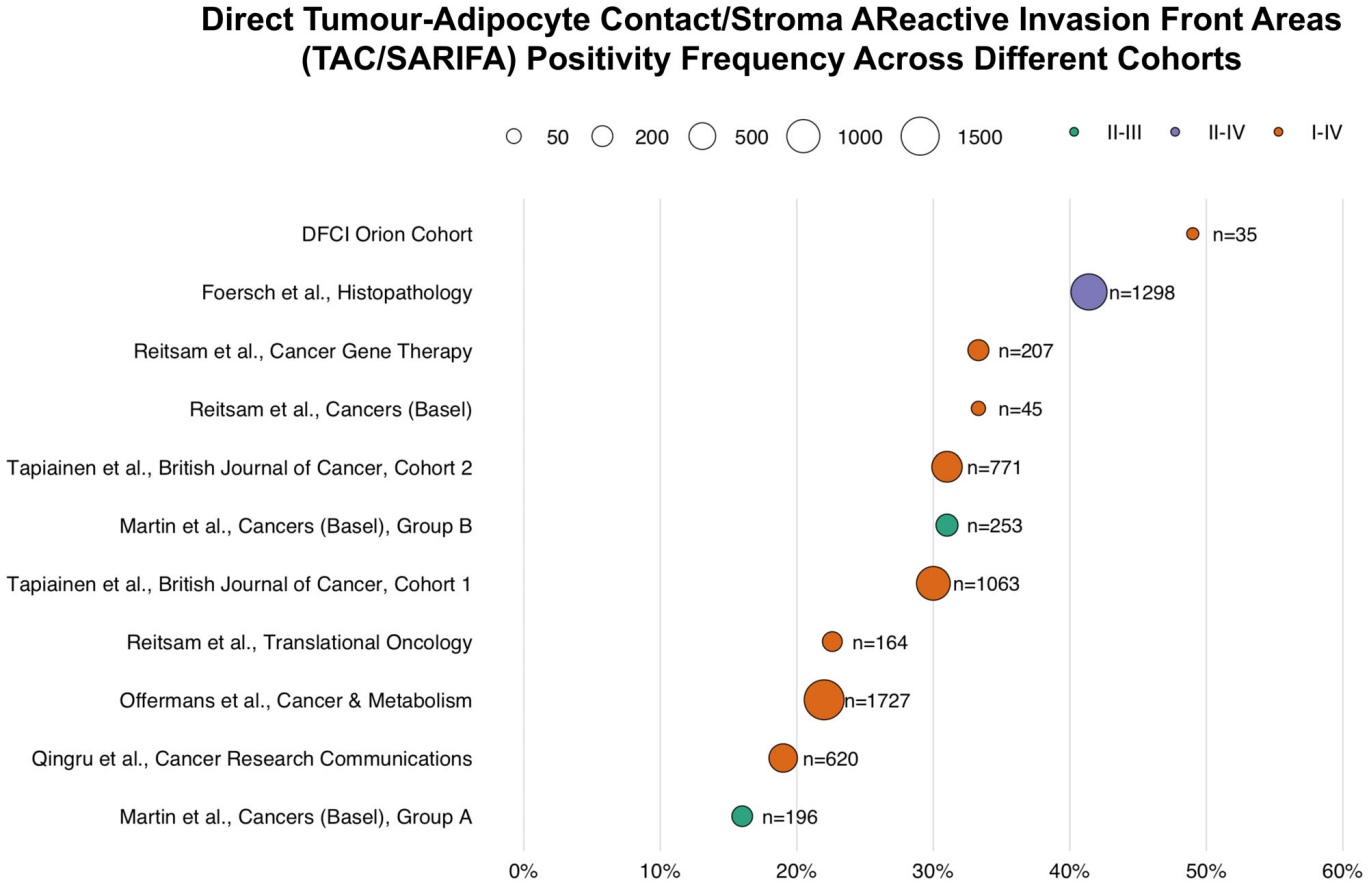
Frequency of TAC/SARIFA positivity across the different studies in CRC. TAC/SARIFA positivity across colorectal cancer cohorts ranged from 16% to 49%, consistent with heterogeneity in cohort collection and tumour stage composition. Each point represents one cohort; point size is proportional to the number of cases scored. Colours indicate reported tumour stages included (green: II‐III, purple: II‐IV, orange: I‐IV). Previous studies used the term SARIFA; we propose the dual terminology tumour–adipocyte contact (TAC)/SARIFA, with TAC being more descriptive of the underlying biological phenomenon.

In some studies, all available tumour slides were assessed, whereas in other studies, only one representative tumour slide was available, leading to similar results. Tapiainen *et al*. could show that assessing TAC/SARIFA status in the slide with the deepest invasion is very reliable.^23^

Characteristics of the currently available studies on TAC/SARIFA in CRC are summarized in Tables 1 and 2.

### Meta‐Analysis: Prognostic Impact of TAC/SARIFA in CRC

In total, seven cohorts (5,932 patients) reported multivariable CSS and five cohorts (4,545 patients) reported multivariable OS, drawn from eight unique cohorts across six studies.^18^, ^20^, ^22^, ^23^, ^25^, ^27^

In random‐effects meta‐analysis, TAC/SARIFA positivity was consistently associated with inferior CSS with a pooled HR of 1.77 (Hartung–Knapp 95% CI 1.55–2.01, *P* < 0.0001) with low between‐cohort heterogeneity (*Q*‐test *P* = 0.49).

Similarly, TAC/SARIFA positivity was associated with an inferior OS with a pooled HR of 1.53 (Hartung–Knapp 95% CI 1.39–1.68, *P* = 0.0002), with again a low heterogeneity (*Q*‐test *P* = 0.76).

In the single available cohorts, TAC/SARIFA positivity was also associated with poorer progression‐free survival (HR 3.56, 95% CI 1.89–6.69, TCGA‐CRC^20^) and recurrence‐free survival (HR 2.60, 95% CI 1.09–6.21, Duesseldorf cohort^27^), and showed an adverse association with metastasis‐free survival (HR 1.50, 95% CI 0.80–2.60, Augsburg Cohort B^25^), but these endpoints were not pooled.

Study weights were primarily driven by the precision of the reported hazard ratios, with the largest and most precise cohort, which was the population‐based Netherlands Cohort Study,^18^ contributing the greatest weight, while smaller cohorts contributed proportionally less. Uncertainty around heterogeneity estimates was wide due to the comparably limited number of cohorts.

Results of the meta‐analyses for CSS and OS are displayed in Figure 3.

**Figure 3:**
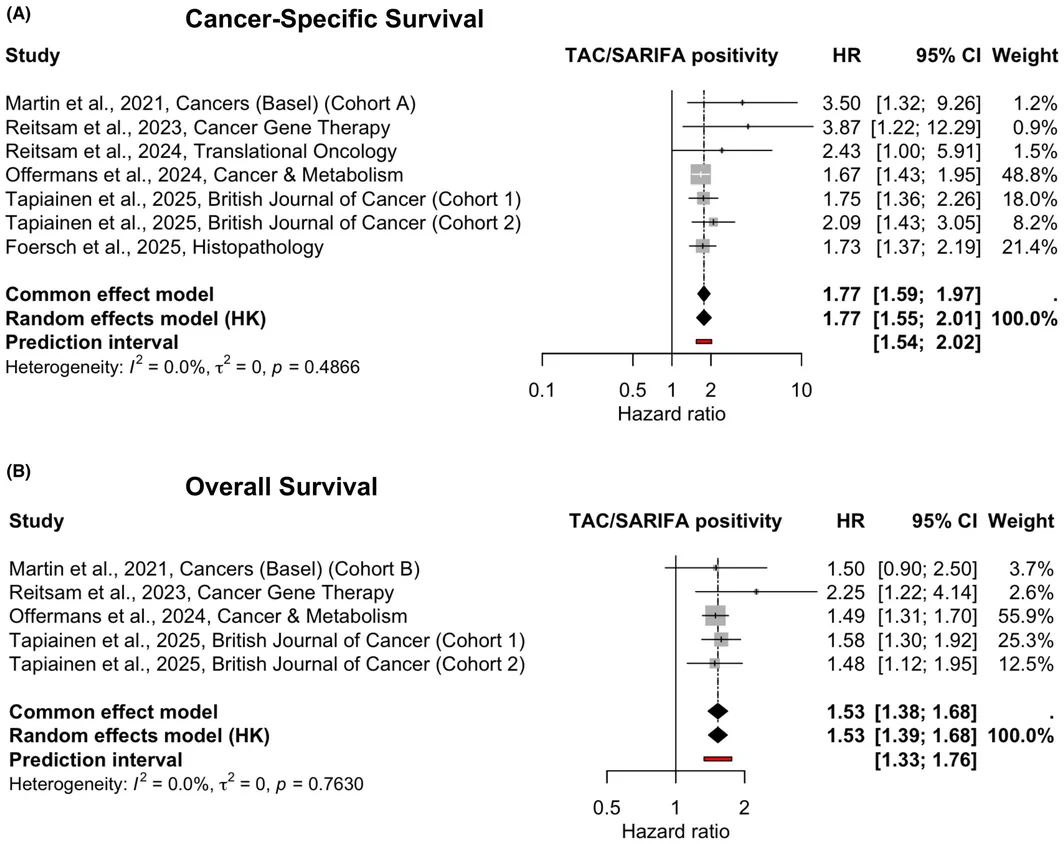
Meta‐analysis of TAC/SARIFA status assessment as prognostic biomarker in CRC. **(A)** TAC/SARIFA positivity is consistently associated with poorer cancer‐specific survival (CSS). **(B)** TAC/SARIFA positivity is consistently associated with poorer overall survival (OS). Forest plots show hazard ratios (HRs) and 95% confidence intervals (CIs) for the association between TAC/SARIFA positivity and CSS (A) and OS (B) across included cohorts. Individual cohort estimates are displayed as squares (with size proportional to inverse‐variance weight) and horizontal lines representing 95% CIs. HRs were derived from multivariable Cox models, with adjustment covariates differing between studies as reported in the original publications. Previous studies used the term SARIFA; we propose the dual terminology tumour–adipocyte contact (TAC)/SARIFA, with TAC being more descriptive of the underlying biological phenomenon.

Study quality was assessed using the NOS for all studies included in the meta‐analysis (Table S2). NOS scores ranged from 7 to 9 out of 9, with all studies rated as good quality according to AHRQ standards. The only study scoring below 9 was the TCGA‐based analysis, which lost points on representativeness (due to case selection constraints inherent to the TCGA dataset) and follow‐up. A descriptive quality assessment covering all 12 publications is provided in Table S3.

### TAC/SARIFA Status Compared to Other Established Biomarkers

TAC/SARIFA has been shown to be of relevance even within subgroup analysis: One study (Offermans*, Reitsam* *et al*.) provided restricted analysis to locally advanced disease, where TAC/SARIFA positivity independently predicted worse outcomes in both pT3 and pT4 tumours.^19^

Another study (Foersch *et al*.) showed that TAC/SARIFA consistently identified patients with poorer prognosis regardless of tumour grade, tumour budding category or histological subtype, including mucinous, micropapillary and serrated adenocarcinomas.^22^

One study reported direct multivariable head‐to‐head models including TAC/SARIFA and budding (Tapianen *et al*., *British Journal of Cancer*, Table S3 ^23^): SARIFA positivity remained an independent predictor of CSS across cohorts, whereas the prognostic effect of tumour budding was attenuated and no longer significant after full adjustment, suggesting that TAC/SARIFA and tumour budding may provide complementary prognostic information at the invasion front.

In the NLCS cohort (Reitsam*, Offermans* et al.), TAC/SARIFA remained independently associated with inferior colorectal CSS and OS, irrespective of *BRAF* or *RAS* mutational status. Importantly, TAC/SARIFA positivity retained prognostic relevance within the pMMR subgroup and across combined *BRAF*/MMR strata, except for *BRAF*‐wild‐type/dMMR tumours.^19^

Finally, stratified analyses in Offermans*, Reitsam* et al. incorporating metabolic phenotypes, demonstrated that the prognostic impact of TAC/SARIFA was largely independent of Warburg subtype based on immunohistochemistry.^18^

### TAC/SARIFA and its Association to Clinicopathological Characteristics and Molecular Subgroups

Across all six studies contributing to the meta‐analysis,^18^, ^20^, ^22^, ^23^, ^25^, ^27^ TAC/SARIFA positivity was statistically significantly (*P* < 0.05) associated with known adverse clinicopathological features, such as locally advanced tumours (pT3/pT4), presence of regional lymph node and distant metastases, and hence higher pTNM stage and higher rate of adjuvant chemotherapy.

TAC/SARIFA positivity is positively correlated with other established histologic risk factors, such as poor differentiation (G3), increased tumour budding (more often Bd3 than SARIFA‐negative^22^ as well as a higher absolute number of tumour buds^16^), lower proportion of tumour (e.g. higher stroma content^27^), as well as non‐mature DR patterns.^32^

In the TCGA dataset, TAC/SARIFA positivity was not linked to a distinct genotype. However, in the large NLCS study, TAC/SARIFA‐positive cases harboured significantly more often *BRAF* mutations (23% TAC/SARIFA‐positive vs 14% TAC/SARIFA‐negative) and were enriched in the *BRAF*‐mutant/MSS cases (16% TAC/SARIFA‐positive vs 6% TAC/SARIFA‐negative).^18^

In terms of transcriptomic subtypes, TAC/SARIFA‐positive CRCs more often belong to the consensus molecular subtypes (CMS) 1 (immune; 24% TAC/SARIFA‐positive vs. 15% TAC/SARIFA‐negative) and 4 (mesenchymal; 53% TAC/SARIFA‐positive vs 31% TAC/SARIFA‐negative) and the pathway‐derived subtype (PDS) 2 (immune and stromal; 45% TAC/SARIFA‐positive vs 21% TAC/SARIFA‐negative).^20^, ^21^

### TAC/SARIFA and Potential Predictive Value

One study revealed, based on the RNA data of the TCGA cohorts, that TAC/SARIFA‐positive CRCs may exhibit distinct gene expression profiles with differential predicted drug sensitivity. Using *oncoPredict* ^37^ to predict therapy response from transcriptomic data, TAC/SARIFA‐positive tumours showed predicted oxaliplatin resistance and dasatinib sensitivity, while no differences were observed for 5‐fluorouracil, irinotecan or lapatinib.^20^ These transcriptomic findings are supported by clinical outcome data in the NLCS demonstrating that patients with TAC/SARIFA‐positive CRC derive a significant CSS and OS benefit from adjuvant (chemo)therapy, whereas this benefit was attenuated or absent in TAC/SARIFA‐negative tumours.^19^

## Discussion

This meta‐analysis and structured review demonstrates that TAC/SARIFA positivity is an independent adverse prognostic factor in CRC. TAC/SARIFA represents a reproducible histomorphological biomarker with good interobserver agreement. Similar to tumour budding, TAC/SARIFA captures tumour–host interactions at the invasion front; however, it captures direct tumour–adipocyte crosstalk rather than EMT. TAC/SARIFA and tumour budding may therefore provide complementary prognostic information at the invasion front. This is supported by TAC/SARIFA's adverse prognostic value across all budding subgroups (Bd1‐3)^22^ but requires further validation.

TAC/SARIFA positivity consistently identifies patients with poorer outcomes irrespective of tumour grade, histological subtype or pT category, including in locally advanced (pT3/4) disease.^18^, ^20^, ^22^, ^23^, ^25^, ^27^ Notably, TAC/SARIFA positivity is predominantly observed at the invasion front within subserosal adipose tissue, resulting in very low positivity rates in early‐stage (pT1/2) tumours (Netherlands Cohort Study 1.2%^18^; TCGA‐CRC 5.8%^20^; Düsseldorf cohort 2.7%^27^; Finnish cohorts 0–1%^23^; Augsburg cohort 6%^16^).

One study demonstrated that TAC/SARIFA may retain valuable prognostic relevance across major molecular strata (*RAS/BRAF* mutation, mismatch repair status),^19^ while another study highlighted its prognostic significance across metabolic phenotypes (Warburg subtype).^18^ If validated in additional prospective cohorts, these findings would support TAC/SARIFA as a marker with prognostic value beyond genomic profiling, underscoring the continuing relevance of conventional H&E histomorphology.

The consistently adverse prognosis suggests that direct tumour–adipocyte interactions reflect biological aggressiveness likely driven by a combination of (i) immune dysregulation,^15^, ^17^, ^20^, ^23^, ^24^, ^31^ (ii) metabolic reprogramming,^15^, ^18^, ^30^, ^31^ (iii) stromal remodelling.^16^, ^20^, ^27^, ^32^ TAC/SARIFA‐positive CRCs are characterized by an immunosuppressive microenvironment (Table 3), which may represent either a predisposing condition or a consequence of direct tumour–host interaction.^38^

**Table 3:** Association between TAC/SARIFA and immunological changes

Study (authors, journal, year)	Methods	Findings
Reitsam *et al. Cancers* (Basel), 2023	Flow cytometry (peripheral blood) CD56 and CD57 immunohistochemistry	Reduction of Natural Killer (NK) cells in TAC/SARIFA‐positive patients: CD56dimCD16bright NK cells and CD56 + CD16+ NK cells
Reitsam *et al. Cancer Gene Therapy*, 2023	RNA sequencing data of the TCGA cohorts, colonic adenocarcinoma (COAD) and rectal adenocarcinoma (READ)	Enrichment of CMS1 subtype in TAC/SARIFA‐positive cases Increased *CD274* gene expression (encoding for PD‐L1)
Reitsam *et al*, *Communications Medicine*, 2024	RNA sequencing data of the TCGA cohorts, colonic adenocarcinoma (COAD) and rectal adenocarcinoma (READ)	Enrichment of PDS2 (immune and stromal) subtype in TAC/SARIFA‐positive cases
Tapiainen *et al. British Journal of Cancer*, 2025	Multiplex and conventional immunohistochemistry combined with digital image analysis	TAC/SARIFA‐positive CRCs with lower CD3+ T cell, CD66b + granulocytes, M1‐like macrophages, and CD14+ HLA‐DR+ mature monocytic cells TAC/SARIFA‐positive CRCs with higher densities of M2‐like macrophages and CD14+ HLA‐DR− immature monocytic cells
Reitsam and Märkl, *British Journal of Cancer* (Correspondence), 2025	Multiplex Immunofluorescence of DFCI/Orion cohort	Lower numbers of FoxP3+ regulatory T cells in TAC/SARIFA‐positive CRCs

CRC, colorectal cancer; SARIFA, Stroma AReactive Invasion Front Areas; TAC, tumour–adipocyte contact.

Transcriptomic data indicate increased lipid metabolism gene expression (especially *FABP4* and *CD36*),^20^ consistent with TAC/SARIFA studies in gastric cancer.^30^, ^31^ In ovarian cancer, adipocyte‐induced FABP4 upregulation drives metastatic progression and chemoresistance, with FABP4 inhibition reducing tumour burden in preclinical models,^39^ highlighting lipid metabolism as a potentially targetable pathway.^40^ TAC/SARIFA formation implies active extracellular matrix degradation, consistent with observed upregulation of matrix proteases in TAC/SARIFA‐positive gastric and colorectal cancers.^16^ While hypothesis‐generating, these findings suggest SARIFA could identify tumours with specific metabolic, stromal or immune vulnerabilities.

Beyond prognosis, emerging data suggest a potential predictive dimension. RNA‐based drug response modelling indicated differential sensitivity in TAC/SARIFA‐positive CRCs, including relative oxaliplatin resistance and increased dasatinib sensitivity^20^ (targeting CMS4‐associated kinases^41^). Preliminary evidence from a single population‐based study suggests that TAC/SARIFA status may be associated with differential benefit from adjuvant therapy, though interaction testing did not reach statistical significance.^19^ These findings remain hypothesis‐generating and require prospective validation.

Independent deep learning (DL) approaches have consistently identified tumour–adipocyte interaction as clinically relevant in CRC, with tumour‐adipose features (TAFs) linked to poor prognosis, lymph node metastasis and adverse outcomes across multicentric cohorts.^42^, ^43^, ^44^ Systematic pathological review demonstrated substantial overlap between DL‐derived TAF and human‐observed TAC/SARIFA, validating tumour–adipocyte interaction as biologically meaningful and clinically relevant.^21^ DL‐derived TAF showed quantity‐dependent outcome associations, with widespread involvement conferring a higher risk than focal disease,^42^ suggesting a possible dose–response relationship for TAC/SARIFA. However, all current TAC/SARIFA studies apply binary classification, which is advantageous for routine pathology given its simplicity, reproducibility and validated prognostic relevance.

Given its definition on routine H&E sections, TAC/SARIFA assessment can be readily integrated into standard histopathological evaluation by trained gastrointestinal pathologists, without additional staining or technical requirements.

The strengths of this work include the structured synthesis of all available TAC/SARIFA studies, the use of adjusted effect estimates and the application of conservative random‐effects meta‐analytic methods with prediction intervals. However, all included studies were retrospective, and differences in cohort composition, slide availability and adjustment strategies cannot be fully excluded. The limited number of studies precludes formal assessment of publication bias and robust pooling of non‐survival endpoints. Validation within large, well‐characterized prospective cohorts and clinical trials will be important to establish TAC/SARIFA's robustness and clinical relevance.

## Conclusion

TAC/SARIFA represents a H&E‐based histomorphological biomarker with consistent adverse prognostic associations in CRC. Prospective studies are needed to determine its clinical utility and potential predictive value for therapeutic decisions.

## Author contributions

NGR conducted the analyses and drafted the initial manuscript. BM, IDN, and MS supervised the work. All authors critically revised the manuscript, provided intellectual input and approved the final version for submission.

## Funding information

NGR is supported by the clinician scientist programme of the Faculty of Medicine, University of Augsburg, Augsburg, Germany.

## Conflicts of interest

NGR has received travel compensation from Bruker Spatial Biology, unrelated to this study.

## Supporting information


**Figure S1.** PRISMA 2020 flow diagram illustrating identification, screening, eligibility assessment, and inclusion of studies evaluating SARIFA in colorectal cancer. The search in PubMed retrieved 17 records, all assessed in full text (3× reviews), 1× on pancreatic adenocarcinoma, 1× on oesophagogastric cancers, 12× on colon cancer/colorectal cancer. Six studies (comprising eight independent CRC cohorts) reported multivariable hazard ratios for time‐to‐event outcomes and were included in the quantitative synthesis. The remaining six reports were excluded from meta‐analysis because they either (i) analysed mixed gastrointestinal tumour entities without CRC‐stratified survival estimates, and/or (ii) focused on biological correlates without reporting multivariable time‐to‐event HRs. These reports were included in the qualitative synthesis. At the time of retrieval, PubMed additionally displayed the review articles, even though not exactly matching the search term, through automatic indexing and relevance matching.
**Table S1**. PRISMA 2020 checklist.
**Table S2**. Newcastle‐Ottawa quality assessment form for cohort studies.
**Table S3**. Descriptive assessment of study quality.

## Data Availability

The data that support the findings of this study are available from the corresponding author upon reasonable request.
