# Supplementary material for: Direct tumour–adipocyte contact / Stroma AReactive Invasion Front Area (TAC/SARIFA) predicts adverse outcome in colorectal cancer: a focused evidence synthesis and meta‐analysis
Source: Histopathology. 2026 Jul 16;89(5):835–48. doi: 10.1111/his.70237 (PMC13643611; doi:10.1111/his.70237)
Supplement: Supplementary file 1 [file HIS-89-835-s001.docx]

# **Supplementary Material**

**Supplementary Figure S1. PRISMA 2020 flow diagram illustrating identification, screening, eligibility assessment, and inclusion of studies evaluating SARIFA in colorectal cancer.** The search in PubMed retrieved 17 records, all assessed in full text (3x reviews, 1x on pancreatic adenocarcinoma, 1x on esophagogastric cancers, 12x on colon cancer/colorectal cancer. Six studies (comprising eight independent CRC cohorts) reported multivariable hazard ratios for time-to-event outcomes and were included in the quantitative synthesis. The remaining six reports were excluded from meta-analysis because they either (i) analysed mixed gastrointestinal tumour entities without CRC-stratified survival estimates, and/or (ii) focused on biological correlates without reporting multivariable time-to-event HRs. These reports were included in the qualitative synthesis. At the time of retrieval, PubMed additionally displayed the review articles, even though not exactly matching the search term, through automatic indexing and relevance matching.

<https://doi.org/10.1002/cl2.1230> PRISMA Flow Diagram Tool

<https://estech.shinyapps.io/prisma_flowdiagram/>

A structured literature search was performed in PubMed on *7th May 2026* using the search terms: (SARIFA OR "Stroma AReactive Invasion Front Areas") AND ("colorectal cancer" OR "colon cancer" OR "rectal cancer" OR "colorectal carcinoma" OR "colon carcinoma" OR "rectal carcinoma"). No restrictions on publication date were applied. The first author, NGR, performed the literature search.

**Supplementary Table S1. PRISMA 2020 Checklist.**

| **Section and Topic** | **Item #** | **Checklist item** | **Location where item is reported** |
| --- | --- | --- | --- |
| **TITLE** | | |  |
| Title | 1 | Identify the report as a systematic review. | Title; identified as "focused evidence synthesis and meta-analysis" |
| **ABSTRACT** | | |  |
| Abstract | 2 | See the PRISMA 2020 for Abstracts checklist. | Abstract; aligned with PRISMA 2020 for Abstracts checklist |
| **INTRODUCTION** | | |  |
| Rationale | 3 | Describe the rationale for the review in the context of existing knowledge. | Introduction, paragraphs 1-3 |
| Objectives | 4 | Provide an explicit statement of the objective(s) or question(s) the review addresses. | Introduction, last paragraph |
| **METHODS** | | |  |
| Eligibility criteria | 5 | Specify the inclusion and exclusion criteria for the review and how studies were grouped for the syntheses. | Methods, 'Literature Identification, Study Selection & Eligibility Criteria'; PICO framework; Supplementary Figure S1 |
| Information sources | 6 | Specify all databases, registers, websites, organisations, reference lists and other sources searched or consulted to identify studies. Specify the date when each source was last searched or consulted. | Methods, 'Literature Identification…'; PubMed, 7 May 2026 |
| Search strategy | 7 | Present the full search strategies for all databases, registers and websites, including any filters and limits used. | Methods, 'Literature Identification…'; full search string provided; no filters or limits applied; authors are familiar with the current literature of the emerging biomarker SARIFA as they established the biomarker |
| Selection process | 8 | Specify the methods used to decide whether a study met the inclusion criteria of the review, including how many reviewers screened each record and each report retrieved, whether they worked independently, and if applicable, details of automation tools used in the process. | Methods, 'Literature Identification…'; screening and data extraction by first author (NGR); eligibility verified by all authors |
| Data collection process | 9 | Specify the methods used to collect data from reports, including how many reviewers collected data from each report, whether they worked independently, any processes for obtaining or confirming data from study investigators, and if applicable, details of automation tools used in the process. | 'Systematic Synthesis…'; data extracted by NGR |
| , Data items | 10a | List and define all outcomes for which data were sought. Specify whether all results that were compatible with each outcome domain in each study were sought (e.g. for all measures, time points, analyses), and if not, the methods used to decide which results to collect. | Methods, 'Systematic Synthesis…'; primary: CSS and OS; secondary: RFS, PFS, MFS (descriptive) |
|  | 10b | List and define all other variables for which data were sought (e.g. participant and intervention characteristics, funding sources). Describe any assumptions made about any missing or unclear information. | Methods, 'Systematic Synthesis…'; cohort characteristics, TAC/SARIFA positivity frequency, covariates in multivariable models extracted per study (Table 1) |
| Study risk of bias assessment | 11 | Specify the methods used to assess risk of bias in the included studies, including details of the tool(s) used, how many reviewers assessed each study and whether they worked independently, and if applicable, details of automation tools used in the process. | Methods, 'Systematic Synthesis…'; Newcastle-Ottawa Scale (NOS) for meta-analysis studies (Suppl. Table S1); descriptive assessment for all 12 publications (Suppl. Table S2); assessed by NGR; formal publication bias assessment not performed (limited number of studies) |
| Effect measures | 12 | Specify for each outcome the effect measure(s) (e.g. risk ratio, mean difference) used in the synthesis or presentation of results. | Methods, 'Systematic Synthesis…'; hazard ratio (HR) with 95% CI |
| Synthesis methods | 13a | Describe the processes used to decide which studies were eligible for each synthesis (e.g. tabulating the study intervention characteristics and comparing against the planned groups for each synthesis (item #5)). | Methods, 'Literature Identification…' and 'Systematic Synthesis…'; studies grouped by endpoint (CSS/OS); only multivariable-adjusted HRs included |
|  | 13b | Describe any methods required to prepare the data for presentation or synthesis, such as handling of missing summary statistics, or data conversions. | Methods, 'Systematic Synthesis…' |
|  | 13c | Describe any methods used to tabulate or visually display results of individual studies and syntheses. | Results; forest plots (Figure 3); Tables 1 and 2 |
|  | 13d | Describe any methods used to synthesize results and provide a rationale for the choice(s). If meta-analysis was performed, describe the model(s), method(s) to identify the presence and extent of statistical heterogeneity, and software package(s) used. | Methods, 'Systematic Synthesis…'; random-effects (REML) with Hartung-Knapp CI; heterogeneity assessed by Q, I², τ²; R meta package |
|  | 13e | Describe any methods used to explore possible causes of heterogeneity among study results (e.g. subgroup analysis, meta-regression). | Methods/Results; Q-test and I² reported; no subgroup analysis performed (limited number of studies) |
|  | 13f | Describe any sensitivity analyses conducted to assess robustness of the synthesized results. | sensitivity analysis substituting Warburg-adjusted model for NLCS cohort (CSS HR 1.76, OS HR 1.51; consistent with primary analysis) |
| Reporting bias assessment | 14 | Describe any methods used to assess risk of bias due to missing results in a synthesis (arising from reporting biases). | Not performed; limited number of eligible studies precludes reliable assessment |
| Certainty assessment | 15 | Describe any methods used to assess certainty (or confidence) in the body of evidence for an outcome. | Not formally assessed, but NOS quality scores reported |
| **RESULTS** | | |  |
| Study selection | 16a | Describe the results of the search and selection process, from the number of records identified in the search to the number of studies included in the review, ideally using a flow diagram. | Results, 'Characteristics of Included Studies'; Supplementary Figure S1 (PRISMA flow diagram) |
|  | 16b | Cite studies that might appear to meet the inclusion criteria, but which were excluded, and explain why they were excluded. | Methods, 'Literature Identification…'; 2 non-CRC records excluded (1 pancreatic, 1 esophagogastric); 3 reviews excluded; 6 CRC studies excluded from meta-analysis with reasons stated |
| Study characteristics | 17 | Cite each included study and present its characteristics. | Presented in Tables 1/2 |
| Risk of bias in studies | 18 | Present assessments of risk of bias for each included study. | Results; NOS scores reported (7–9/9, all good quality); Supplementary Table S1 |
| Results of individual studies | 19 | For all outcomes, present, for each study: (a) summary statistics for each group (where appropriate) and (b) an effect estimate and its precision (e.g. confidence/credible interval), ideally using structured tables or plots. | Results; forest plots (Figure 3A, 3B); HRs with 95% CIs for each cohort |
| Results of syntheses | 20a | For each synthesis, briefly summarise the characteristics and risk of bias among contributing studies. | Results, 'Meta-analysis…'; all studies retrospective, NOS 7–9/9 (good quality) |
|  | 20b | Present results of all statistical syntheses conducted. If meta-analysis was done, present for each the summary estimate and its precision (e.g. confidence/credible interval) and measures of statistical heterogeneity. If comparing groups, describe the direction of the effect. | Results, 'Meta-analysis…'; CSS HR 1.77 (95% CI 1.55-2.01); OS HR 1.53 (95% CI 1.39-1.68) |
|  | 20c | Present results of all investigations of possible causes of heterogeneity among study results. | Results; Q-test p=0.49 (CSS), p=0.76 (OS); I²=0% for both; no formal subgroup analysis (limited studies) |
|  | 20d | Present results of all sensitivity analyses conducted to assess the robustness of the synthesized results. | Warburg-adjusted sensitivity: CSS HR 1.76, OS HR 1.51 (consistent with primary) |
| Reporting biases | 21 | Present assessments of risk of bias due to missing results (arising from reporting biases) for each synthesis assessed. | Not assessed; stated in Methods (limited number of studies) |
| Certainty of evidence | 22 | Present assessments of certainty (or confidence) in the body of evidence for each outcome assessed. | Discussion; limitations of retrospective design and limited number of studies discussed |
| **DISCUSSION** | | |  |
| Discussion | 23a | Provide a general interpretation of the results in the context of other evidence. | Discussion. |
|  | 23b | Discuss any limitations of the evidence included in the review. | Discussion, final paragraph. |
|  | 23c | Discuss any limitations of the review processes used. | Discussion, final paragraph. |
|  | 23d | Discuss implications of the results for practice, policy, and future research. | Discussion and conclusion. |
| **OTHER INFORMATION** | | |  |
| Registration and protocol | 24a | Provide registration information for the review, including register name and registration number, or state that the review was not registered. | Methods, 'Literature Identification…'; not registered |
|  | 24b | Indicate where the review protocol can be accessed, or state that a protocol was not prepared. | No protocol was prepared; stated in Methods |
|  | 24c | Describe and explain any amendments to information provided at registration or in the protocol. | Not applicable (no protocol/registration) |
| Support | 25 | Describe sources of financial or non-financial support for the review, and the role of the funders or sponsors in the review. | Acknowledgements; NGR supported by clinician scientist programme, University of Augsburg; no external funding for the review |
| Competing interests | 26 | Declare any competing interests of review authors. | Acknowledgements; NGR received travel compensation from Bruker Spatial Biology (unrelated) |
| Availability of data, code and other materials | 27 | Report which of the following are publicly available and where they can be found: template data collection forms; data extracted from included studies; data used for all analyses; analytic code; any other materials used in the review. | R analysis code available upon request |

*From:*  Page MJ, McKenzie JE, Bossuyt PM, Boutron I, Hoffmann TC, Mulrow CD, et al. The PRISMA 2020 statement: an updated guideline for reporting systematic reviews. BMJ 2021;372:n71. doi: 10.1136/bmj.n71. This work is licensed under CC BY 4.0. To view a copy of this license, visit <https://creativecommons.org/licenses/by/4.0/>

| **Supplementary Table S2. Newcastle-Ottawa Quality Assessment Form for Cohort Studies.** | | | | | |
| --- | --- | --- | --- | --- | --- |
| **Study** | **Selection** | **Comparability** | **Outcome** | **Total Stars & AHRQ Standards** |  |
| Martin et al, Cancers, 2021 | 4 stars | 2 stars | 3 stars | total: 9, good quality |  |
| Reitsam et al, Cancer Gene Therapy, 2023 | 3 stars | 2 stars | 2 stars | total: 7, good quality |  |
| Reitsam et al, Translational Oncology, 2024 | 4 stars | 2 stars | 3 stars | total: 9, good quality |  |
| Offermans*, Reitsam* et al, Cancer Metabolism, 2024 | 4 stars | 2 stars | 3 stars | total: 9, good quality |  |
| Tapiainen et al, British Journal of Cancer, 2025 | 4 stars | 2 stars | 3 stars | total: 9, good quality |  |
| Foersch et al, Histopathology, 2025 | 4 stars | 2 stars | 3 stars | total: 9, good quality |  |

**Supplementary Table S3. Descriptive Assessment of Study Quality**

| Study | Design/representativeness | TAC/SARIFA definition | Outcome definition | Confounder adjustment |
| --- | --- | --- | --- | --- |
| Martin et al, Cancers, 2021 | some limitations (single centre; stage II–III) | no obvious limitations | no obvious limitations | no obvious limitations |
| Reitsam et al, Cancers, 2023 | *for biological exploration* | no obvious limitations | NA | NA |
| Reitsam et al, Cancer Gene Therapy, 2023 | some limitations (TCGA; selected cases with representative WSI + RNA) | some limitations (TCGA constraints; all slides with only superficial tissue were excluded, thorough histopathological review) | no obvious limitations | no obvious limitations |
| Hu et al, Cancer Research Communications, 2023 | *no prognostic data; for biological exploration* | no obvious limitations | NA | NA |
| Reitsam et al, Translational Oncology, 2024 | some limitations (single centre) | no obvious limitations | no obvious limitations | no obvious limitations |
| Reitsam et al, Communications Medicine, 2024 | *for association between deep learning-derived tumor adipose feature (TAF) and human-derived TAC/ TAC/SARIFA* | no obvious limitations | no obvious limitations (TCGA cohort) | NA (no multivariate analysis has been performed here) |
| Offermans*, Reitsam* et al, Cancer Metabolism, 2024 | no obvious limitations (population-based) | no obvious limitations | no obvious limitations | no obvious limitations |
| Tapiainen et al, British Journal of Cancer, 2025 | no obvious limitations (two centres; large) | no obvious limitations | no obvious limitations | no obvious limitations |
| Reitsam et al, British Journal of Cancer, 2025 | notable limitations (no HR reported, only for biological exploration relevant) | no obvious limitations | no obvious limitations (DFCI Orion cohort) | NA (no multivariate analysis has been performed here) |
| Reitsam*, Offermans*, American Journal of Surgical Pathology, 2025 | no obvious limitations(population-based) | no obvious limitations | no obvious limitations | no obvious limitations |
| Foersch et al, Histopathology, 2025 | no obvious limitations (3 centres; retrospective) | no obvious limitations | no obvious limitations | no obvious limitations |
| Reitsam*, Grosser* et al, Pathobiology, 2026 | some limitations (single centre; n=96) | no obvious limitations | no obvious limitations | NA (no multivariate analysis has been performed here) |
